# Negative Clinical Evolution in COVID-19 Patients Is Frequently Accompanied With an Increased Proportion of Undifferentiated Th Cells and a Strong Underrepresentation of the Th1 Subset

**DOI:** 10.3389/fimmu.2020.596553

**Published:** 2020-11-26

**Authors:** Juan Francisco Gutiérrez-Bautista, Antonio Rodriguez-Nicolas, Antonio Rosales-Castillo, Pilar Jiménez, Federico Garrido, Per Anderson, Francisco Ruiz-Cabello, Miguel Ángel López-Ruz

**Affiliations:** ^1^Servicio de Análisis Clínicos e Inmunología, Hospital Universitario Virgen de las Nieves, Granada, Spain; ^2^Servicio de Medicina Interna, Hospital Universitario Virgen de las Nieves, Granada, Spain; ^3^Instituto de Investigación Biosanitaria de Granada (ibs.GRANADA), Granada, Spain; ^4^Departamento Bioquímica, Biología Molecular e Inmunología III, University of Granada, Granada, Spain; ^5^Servicio de Enfermedades Infecciosas, Hospital Universitario Virgen de las Nieves, Granada, Spain; ^6^Departamento de Medicina, University of Granada, Granada, Spain

**Keywords:** T-CD8 lymphocytes flow cytometric immunophenotyping, Th lymphocytes, T cell response, severe acute respiratory syndrome coronavirus 2, severe coronavirus disease 2019

## Abstract

The severity of SARS-CoV-2 infection has been related to uncontrolled inflammatory innate responses and impaired adaptive immune responses mostly due to exhausted T lymphocytes and lymphopenia. In this work we have characterized the nature of the lymphopenia and demonstrate a set of factors that hinder the effective control of virus infection and the activation and arming of effector cytotoxic T CD8 cells and showing signatures defining a high-risk population. We performed immune profiling of the T helper (Th) CD4+ and T CD8+ cell compartments in peripheral blood of 144 COVID-19 patients using multiparametric flow cytometry analysis. On the one hand, there was a consistent lymphopenia with an overrepresentation of non-functional T cells, with an increased percentage of naive Th cells (CD45RA+, CXCR3-, CCR4-, CCR6-, CCR10-) and persistently low frequency of markers associated with Th1, Th17, and Th1/Th17 memory-effector T cells compared to healthy donors. On the other hand, the most profound alteration affected the Th1 subset, which may explain the poor T cells responses and the persistent blood virus load. Finally, the decrease in Th1 cells may also explain the low frequency of CD4+ and CD8+ T cells that express the HLA-DR and CD38 activation markers observed in numerous patients who showed minimal or no lymphocyte activation response. We also identified the percentage of HLA-DR+CD4+ T cells, PD-1+CD+4/CD8+ T cells in blood, and the neutrophil/lymphocyte ratio as useful factors for predicting critical illness and fatal outcome in patients with confirmed COVID-19.

## Introduction

The novel severe acute respiratory syndrome coronavirus 2 (SARS-CoV-2) is the cause of the coronavirus disease 2019 (COVID-19) pandemic that emerged in Wuhan (China) in early December 2019 (1). On the 30^th^ of January 2020, the world health organization declared the SARS-CoV-2 outbreak an international health emergency and 9 months later more than 41,000,000 infected have been reported worldwide, with more than 1,125,000 deaths (2).

SARS-CoV-2 belongs to the betacoronavirus (β-CoVs) genus, as do the SARS-CoV and MERS-CoV (3). It is a zoonotic virus whose possible reservoirs are bats and/or pangolins (4). Phylogenetic analysis showed that SARS-CoV-2 is closely related to a bat coronavirus but also has sequence identity to SARS-CoV and MERS-CoV (5). Like SARS-CoV, SARS-CoV-2 uses angiotensin-converting enzyme 2 (ACE2) as a receptor for entry into the cells, infecting type II pneumocytes of the lung epithelium (6, 7). ACE2 is also expressed in the upper epithelium of the esophagus, ileum and colon enterocytes, myocardial cells, cells of the proximal kidney tubule, bladder urothelium, and the oral mucosa (8).

The SARS-CoV-2 infection is characterized by cough, fever, dyspnea, myalgia, rhinorrhea, diarrhea, and conjunctivitis (9, 10). Most cases (80–90%) are mild or asymptomatic, while 10% can develop severe disease (9). The most severe cases suffer unilateral or bilateral pneumonia and acute respiratory distress syndrome (ARDS), shock and multi-organ failure which might result in death (9). The mortality rate of the disease is around 3% (11). There are several factors that influence the risk of intensive care unit (ICU) admission or death, including advanced age, previous pathologies, and overweight (12).

Regarding clinical and biochemical parameters, COVID-19 patients present an elevation of proinflammatory cytokines like interleukin-1 (IL-1), IL-6, tumor necrosis factor alpha (TNF- α), interferon gamma (IFN-γ), C-X-C motif chemokine ligand 10 (CXCL10), and monocyte chemoattractant protein-1 (MCP-1), which can lead in certain cases to a cytokine storm (11, 12). The cytokine storm is one of the main reasons for the development of ARDS and multi-organ failure (13). In addition to cytokines, there are several inflammation and coagulation parameters that are elevated in these patients, such as C-reactive protein (CRP), ferritin, lactate dehydrogenase (LDH) D-dimer, and fibrinogen (12). Furthermore, lymphopenia has been observed in 85% of hospitalized patients (14) resulting in a worse prognosis (15).

Lymphopenia as an effect produced by SARS-CoV-2 infection has been reported in numerous works (16). However, no studies have specifically investigated functional Th subtypes or which specific subpopulation is affected by the lymphopenia. This information is valuable because it will clarify the nature of the compromised immunology response, and could provide a rationale for immune restorative treatments. Likewise, an answer to the cause of lymphopenia has not been found, although possible reasons have been described (17).

Our work is based on the clinical data of 145 COVID-19 patients admitted to the University Hospital Virgen de las Nieves (Granada), Spain. We have compared the immunological profile in peripheral blood between three groups of patients: asymptomatic, hospitalized, and patients admitted to the ICU. We performed a flow cytometric analysis of the different subpopulations of T lymphocytes [CD4, CD8, Th1, Th2, Th17, Th22, regulatory T cells (Treg), and T follicular helper cells (TFH)] at the time of admission, as well as activation and exhaustion markers [HLA-DR, CD38, CD39, programmed death 1 (PD-1) and T cell immunoreceptor with Ig and ITIM domains (TIGIT)], looking for which subpopulations are affected by the lymphopenia and their relationship with the different clinical, biochemical and hospital stay parameters, as well as the different effects that SARS-CoV-2 infection can generate on the immune response.

In this work, we show for the first time a profound effect of a SARS-CoV-2 infection on the Th1 component. The fact that these cells are important in the control of the response mediated by CD8+ T cells through the production of IL-2 and IFN-γ (18), makes us believe that our findings are relevant, since they can generally explain the poor T cell response and the prolonged viremia in COVID-19 patients.

## Methods

### Samples

Patients (N=144) diagnosed with COVID-19 admitted to University Hospital Virgen de las Nieves, Granada, Spain, were prospectively included in our study between March 2020 and June 2020 in order to conduct an observational study. The patients are distributed as follows.

A first cohort, “non-ICU hospitalized patients” was composed by one hundred patients recruited within 24 h of hospital admission. Peripheral blood was collected at enrolment. In this group the median age was 74.5 years and 51.0% were females. This cohort was composed of elderly patients, with a high incidence of cardiovascular diseases, with high blood pressure being the most common comorbidity, affecting more than half of the patients. Fourteen percent of our patients had a history of cerebrovascular disease, and 12% had a previous myocardial infarction. Approximately a quarter of patients were diabetic and 15% suffered from chronic kidney disease. Most of the patients had no other pre-existing pulmonary condition. Mean follow-up time for hospitalized patients was 12.5 (9.8–15.3) days. During the hospital stay, twenty patients (19.6%) from this group died and four (3.9%) needed transfer to the intensive care unit (ICU). From seven patients of this group, a second sample was collected 70 days after hospital admission for the realization of a longitudinal study.

A second cohort, “ICU hospitalized patients” was composed by an independent group of 17 ICU COVID-19 patients, composed mostly of men (76.5%) with a median age of 69 years. This group showed a high incidence of cardiovascular diseases, being hypertension the most common comorbidity, affecting 53% of the ICU patients. Diabetes mellitus was the second most common comorbidity among ICU patients, affecting approximately one third of them. The median length of admission in this group exceeded 2 months. Mortality in this group was 11.8%.

The third cohort “asymptomatic recovered donors” was composed by a group of 27 hospital staff members with no previous symptoms that tested positive for IgG against SARS-CoV-2. This group was composed of 81.5% (22) females and 18.5% (5) males, with a median age of 43 (34.0–58.0) years.

The control group comprised 42 healthy blood donors recruited among hospital staff tested negative for SARS-CoV-2, with a median age of 61 years (55–62), 90.5% (38) females and 9.5% (4) males.

Peripheral blood was collected from all subjects. We followed the patients until discharge or death, collecting data about clinical manifestations, laboratory data, and demographic data. Sequential Organ Failure Assessment Score (SOFA score), that integrates data from cardiovascular, respiratory, hepatic, coagulation, neurological, and renal systems (19), was calculated by trained physicians at admission. **Supplementary Table 1** provides a summary of the demographic and clinical features of patients. All individuals were natives from the Granada area.

All patient samples were collected according to the local medical ethics regulations, after informed consent was obtained by the subjects, their legal representatives, or both, according to the Declaration of Helsinki. The study was approved by the local ethics committee (Cod. 0766-N-20).

### Statistical Analysis

Categorical data were described as percentages, and non-categorical data were expressed as median and quartile intervals. The parametric Student’s t test was used to compare groups when the distribution was normal (as checked by the Kolmogorov-Smirnov test) and the non-parametric Mann–Whitney U test when it was not. Spearman analysis was used to evaluate correlations between quantitative variables. Fisher’s exact test was used to determine if there were associations between two categorical variables. The R function *ggcorrplot* was used to calculate and visualize correlations between variables, displaying the positive correlations in red and negative correlations in blue. Correlations with P-values > 0.05 were considered as insignificant and left blank. To evaluate the capacity to predict mortality using cytometry and biochemical parameters measured at hospital admission, we plotted the receiver operating characteristics (ROC) curve, and calculated the area under the receiver operating characteristic (AUC).

SPSS statistical software (Windows version 20, IBM, Armonk, NY, USA) was used for statistical analysis. To compute and visualize the correlation matrix R package *ggcorrplot* was used. The P-values are not corrected and it was considered a type 1 error (α) of 0.05 to reject the hypothesis testing.

### Immune Characterization by Cytometry

**Figure 1:**
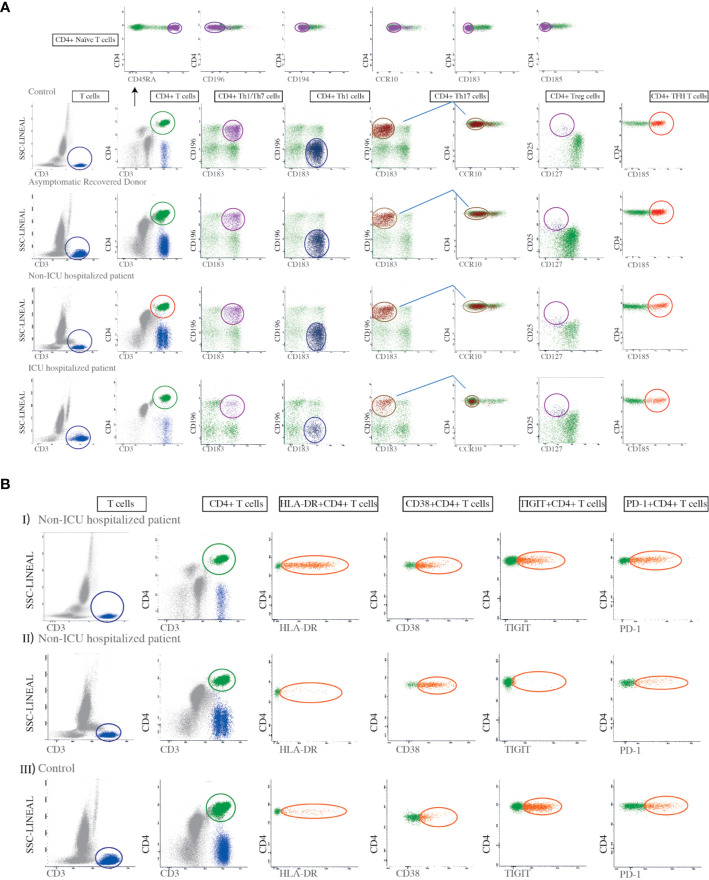
Gating strategies for the identification of Th subsets and the evaluation of T cell activation and exhaustion markers. **(A)** First a lymphocyte gate was defined based on high CD3 expression and low side-scatter (SSC) complexity. A minimum of 200,000 events/sample were collected in this gate. Second, the cells in the lymphocyte gate were divided into CD4+ and CD8-lymphocytes. Third, we analyzed the CD4+ lymphocytes for the expression of CD183, CD196, and CCR10 in order to identify Th1 (CD4+CD183+CD196-), Th1/Th17 (CD4+CD196+CD183+), and Th17 cells (CD4+CD183-CD196+CCR10-). A human regulatory T cell (Tregs) cocktail was used to identify regulatory T cell subsets defined as CD4+CD25brightCD127low. This gating strategy was applied to all controls, asymptomatic recovered donors, non-ICU hospitalized patients, and ICU hospitalized patients, as indicated in the figure. For the identification of naïve CD4+ T cells we used antibodies against CD45RA, CD183, CD185, CD186, CD194, CD196, and CCR10, selecting those that were bright for CD45RA but negative for the rest of the mentioned markers, as shown in the top panel. The amount of each T cell subset was expressed as a percentage of total CD4+ cells. **(B)** Dot plots showing the expression of the HLA-DR, CD38, TIGIT, and PD-1 markers on CD4+ T cells from two patients with different degrees of HLA-DR expression, in addition to a control. Panel I shows a non-ICU hospitalized patient with high expression of HLA-DR on CD4+ T cells while II shows another non-ICU hospitalized patient with low expression of HLA-DR on CD4 + T cells. Panel III shows a control.

Whole peripheral blood (PB) samples were stained for cell surface markers using a direct immunofluorescence technique. Eight-color combinations of monoclonal antibodies (Mab) were used to identify the different T cell subsets. CD3 cell subpopulations were determined by the selection of CD45+, CD3+ cells in the lymphocyte gate. The Th1, Th2, Th1/Th17, Th17, Th22, and TFH subpopulations were detected in the CD4+ plot, based on the expression levels of CXC chemokine receptor 3 (CXCR3), CXC chemokine receptor 5 (CXCR5), CD194, CCR4, CD196, and chemokine receptor 6 (CCR6) (**Figure 1A**). A human regulatory T cell cocktail was used to identify Tregs, defined as CD127low-CD25bright-CD4+. Naïve CD4+ T cells were detected by bright expression of CD45RA and negative expression for all other chemokine receptors (**Figure 1A**). The amount of Tregs was expressed as a percentage of total CD4+ cells. All Mabs were purchased from BD Biosciences, San Diego, CA. CD4+ T cell subsets were defined by chemokine receptors expression according to **Supplementary Table 3**.

The Mab combinations for the detection of the different Th subsets were based on the panels used by EuroFlow-IMM TCD4 (20). T cell activation-associated markers (HLA-DR, and CD38) and T cell exhaustion markers (TIGIT and PD-1) were also examined (**Figure 1B**). The specificity and fluorochromes of each reagent used are listed in **Supplementary Table 2**. Stained cell suspensions were analyzed on a FACSCanto II flow cytometer (BD Biosciences, San José, CA, USA). An average of 300,000 events per tube corresponding to the whole PB cellularity was acquired. The Infinicyt^TM2^2.0 software was employed for multiparametric analysis. For instrument set up, BD one flow set up standard operating procedures were used. T cells were selected in a SSC versus CD3 bivariate dot plot histogram after exclusion of debris.

## Results

### Clinical Inflammatory Syndrome in COVID-19 Patients

Most COVID-19 patients presented at the time of hospital admission a clinical inflammatory syndrome, characterized by the elevation of several biochemical inflammatory markers.

In 93% of the non-ICU hospitalized patients, fibrinogen was raised. The levels of C reactive protein (CRP), lactate dehydrogenase (LDH), and D-dimer (a fibrin degradation product) were above normal range in 89, 85, and 75% of the non-ICU hospitalized patients, respectively. Two thirds of the non-ICU hospitalized patients showed an elevation of ferritin whereas troponin I was above normal range in 57%. Brain natriuretic peptide (BNP) was elevated in 53% of tested non-ICU hospitalized patients and procalcitonin was raised in 32%.

A total of 41 patients, were tested for IL-6 (30 non-ICU hospitalized patients and eleven ICU hospitalized patients). Twenty-three non-ICU hospitalized patients and all except one ICU hospitalized patients showed increased levels for IL-6 in serum. Additional analysis showed that IL-6 is positively correlated with Th17 (*P*=0.014), CRP (*P*<0.001), and fibrinogen (*P*=0.008).

All the ICU hospitalized patients had above normal levels of CRP, LDH, ferritin, troponin I, D-dimer, and fibrinogen, whereas procalcitonin was elevated in the 88% and BNP in 41%.

### Changes in Leukocyte Populations in Peripheral Blood From COVID-19 Patients

Non-ICU hospitalized patients were observed to have an increased leukocyte count compared to controls. This was also observed in non-ICU hospitalized patients (**Table 1**). Though most patients had white blood cell counts (WBC) in the normal range, 23% of non-ICU hospitalized patients and 23.5% of ICU hospitalized patients showed clinical leukocytosis. There were no significant intra-group differences associated with sex in leukocyte populations.

**Table 1:** Absolute count and percentages of the different cell populations in peripheral blood samples of healthy controls and coronavirus disease 2019 (COVID-19) patients.

	Controls (n=42)	Non-ICU hospitalized patients (n=100)	ICU hospitalized patients (n=17)	Asymptomatic recovered donors (n=27)	P_1_	P_2_	P_3_	P_4_	P_5_
WBC (×10^−3^/ml)	5,970 (5,200–5970)	6,680 (4,810–9,960)	7250 (4,010–10,140)	6,520 (5,000–8,610)	n.s	n.s	n.s	n.s	n.s
Lymphocyte pool (×10^−3^/ml)	2,080 (1,740–2,430)	1,120 (760–1,580)	990 (680–1,610)	2,150 (1,700–2,510)	**4.2x10^−11^**	**1.28x10^−04^**	n.s	n.s	**2.60x10^−04^**
T cells (×10^−3^/ml)	1,216 (1,006–1,707)	859 (549–1,248)	630 (500–876)	1,482 (1,075–1,689)	**1.22x10^−05^**	**7.23x10^−06^**	n.s	n.s	**3.91x10^−06^**
T-CD4+	773 (616–1,075)	387 (258–573)	313 (262–467)	853 (627–1,044)	**2.28x10^−11^**	**3.22x10^−07^**	n.s	n.s	**1.05x10^−06^**
T-CD8+	330 (213–469)	172 (101–303)	167 (123–334)	337 (274–614)	**2.27x10^−07^**	**0.007**	n.s	n.s	**0.001**
**T lymphocytes (%)**									
CD3	20.34 (16.34–23.34)	8.60 (4.72–12.96)	8.90 (4.03–15.72)	22.62 (16.84–24.93)	**8.61x10^−14^**	**5.73x10^−06^**	n.s	n.s	**9.73x10^−06^**
CD4	12.46 (10.88–16.00)	5.41 (3.00–8.76)	4.32 (2.68–9.78)	11.93 (10.26–15.72)	**2.62x10^−14^**	**1.74x10^−06^**	n.s	n.s	**5.43x10^−05^**
CD8	5.44 (3.89–7.64)	2.24 (1.40–4.38)	3.01 (1.59–4.75)	6.51 (4.92–8.91)	**1.58x10^−08^**	**1.68x10^−03^**	n.s	n.s	**6.53x10^−04^**
**Ratio T cells**									
CD4/CD8	2.40 (1.85–3.40)	2.41 (1.50–3.53)	1.96 (1.04–3.03)	2.06 (1.48–2.82)	n.s	n.s	n.s	n.s	n.s
**CD4 T lymphocytes (%)**									
Th1	33.00 (27.49–38.10)	24.73 (17.32–31.50)	27.88 (20.72–36.19)	28.33 (24.82–34.62)	1.53x10^−06^*	n.s	n.s	n.s	n.s
Th17	10.52 (8.64–13.06)	9.91 (7.59–13.53)	11.64 (9.48–14.71)	9.62 (6.98–11.31)	n.s	n.s	n.s	0.040*	0.026*
Th1/Th17	7.93 (6.56–10.58)	5.62 (3.84–7.60)	6.93 (5.49–10.23)	7.96 (6.10–10.15)	7.84x10^−07^*	n.s	n.s	n.s	n.s
Treg	7.23 (5.99–8.73)	6.20 (4.94–7.90)	7.49 (6.26–9.47)	5.90 (7.16–8.39)	n.s	n.s	n.s	**0.024**	n.s
Th2	5.93 (4.19–8.60)	7.47 (5.72–8.79)	8.51 (6.25–9.89)	7.06 (5.57–8.66)	n.s	**0.023**	n.s	n.s	n.s
Th22	0.70 (0.42–.1.18)	1.17 (0.72–1.78)	0.54 (0.41–0.93)	1.59 (0.50–6.12)	8.18x10^−05^*	0.001*	**0.005**	**2.69x10^−04^**	**0.015**
TFH	22.99 (21.05–27.80)	18.52 (13.04–25.40)	18.52 (14.60–21.88)	24.53 (18.45–30.42)	**0.001**	0.001*	n.s	n.s	**0.004**
PD–1+CD4+	32.54 (23.62–40.07)	26.45 (18.20–39.80)	35.76 (27.27–42.24)	25.40 (11.05–39.36)	**0.033**	n.s	n.s	n.s	n.s
TIGIT+CD4+	20.28 (14.32–26.10)	13.55 (8.57–18.10)	18.81 (14.22–24.75)	16.12 (10.69–23.55)	5.52x10^−04^*	n.s	n.s	**0.009**	n.s
HLA-DR+CD4+	11.73 (9.65–15.58)	14.38 (9.46–20.10)	14.75 (10.73–23.11)	9.46 (7.84–12.38)	n.s	n.s	**0.017**	n.s	**0.004**
CD39+CD4+	4.41 (1.56–6.48)	4.72 (1.93–8.02)	ND	ND	n.s	NA	NA	n.s	n.s
CD38+CD4+	5.78 (4.35–6.93)	4.66 (3.50–6.71)	6.32 (2.44–9.81)	4.97 (4.21–5.92)	**0.028**	n.s	n.s	n.s	n.s
CD4+ naïve T cells	25.60 (19.40–33.70)	33.37 (20.91–43.59)	36.94 (32.15–50.21)	ND	0.022*	0.001*	NA	NA	NA
**CD8 T lymphocytes (%)**									
PD-1+CD8+	38.44 (27.95–46.19)	32.50 (19.24–42.80)	45.83 (29.91–55.99)	27.31 (16.55–34.61)	0.043*	n.s	**0.006**	**0.004**	**0.002**
TIGIT+CD8+	40.57 (26.36–55.72)	28.81 (14.54–39.64)	30.28 (21.58–46.90)	31.25 (20.69–39.91)	0.001*	n.s	0.014*	n.s	n.s

Results are expressed as median (Q_1_–Q_3_). Mann–Whitney U test are displayed. P values indicated in bold are statistically significant. P_1_ value: statistic comparison between non-ICU hospitalized patients and the control group. P_2_ value: statistic comparison between ICU patients and the control group. P_3_ value: statistic comparison between asymtomatic recovered donors and the control group. P_4_ value: statistic comparison between ICU patients and non-ICU hospitalized patients. P_5_ value: statistic comparison between ICU patients and asymtomatic recovered donors. *Statistical analysis was evaluated by Student’s t test. WBC, white blood cells; NA, not analyzed; n.s, not significant.

Increased neutrophil numbers were observed in non-ICU hospitalized patients and ICU hospitalized patients compared to healthy controls (**Table 1**). Furthermore, 25% of the non-ICU hospitalized patients and 17% of the ICU hospitalized patients had severe neutrophilia.

We also observed a significantly lower lymphocyte count in COVID-19 patients compared to healthy controls (**Table 1**). Besides, 49% of non-ICU hospitalized patients, and 65% of ICU hospitalized patients exhibited lymphopenia.

To assess the impact of acute SARS-CoV2 infection on T cell populations, we performed an in depth immunophenotypic analysis of the activation status and differentiation of functional T cell populations in peripheral blood of healthy donors, non-ICU hospitalized patients, ICU hospitalized patients, and asymptomatic recovered donors using flow cytometry. We could not detect significant intra-groups differences between sexes. A decrease in cell count was observed for CD4+ and CD8+ T cells in non-ICU hospitalized patients and in ICU hospitalized patients. The reduction in CD4+ and CD8+ T cells was equally pronounced. Indeed, there were no significant differences in the CD4:CD8 ratios between groups (**Table 1**).

Interestingly, we found that the percentages of Th1, Th1/Th17, and TFH cells were significantly reduced in non-ICU hospitalized patients with respect to healthy donors (**Table 1**). The reduction of Th1 cells was especially pronounced, decreasing by almost 10% in non-ICU hospitalized patients. In contrast, the percentage of Th22 cells in non-ICU hospitalized patients was slightly elevated compared to healthy donors (**Table 1**). The decrease in Th17 and Tregs cells, although not statistically significant when comparing percentages, was significant when comparing cell counts. Conversely, naïve CD4+ T cells were greatly overrepresented in non-ICU hospitalized patients and ICU hospitalized patients (**Table 1**).

In ICU hospitalized patients, albeit the T lymphocyte pool was more depleted compared to the other group of patients, only the TFH population was significantly reduced, whereas the reduction in Th1, Th17, and Th1/Th17 percentages was less pronounced, not reaching significance. In contrast to the non-ICU hospitalized patients, the Th22 population was significantly reduced in ICU hospitalized patients compared to healthy controls (**Table 1**). However, absolute cell counts of Th1, Th17, Th1/Th17, TFH, and Treg cells were significantly reduced compared to healthy donors. Furthermore, naïve CD4+ T cells were also notably elevated in this group (**Table 1**).

Asymptomatic recovered donors showed normal values in CD4+ T cells subpopulations, being only Th22 cells slightly elevated with respect to healthy donors.

Concerning T cell exhaustion markers, non-ICU hospitalized patients presented a significant decrease in PD-1+CD4+, and TIGIT+CD4+, PD-1+CD8+, and TIGIT+CD8+ populations. Noticeably, asymptomatic recovered donors still showed decreased levels of PD-1+CD8+ and TIGIT+CD8+ cell populations (**Table 1**).

### Changes in Lymphocyte Subpopulations After Recovery

**Figure 2:**
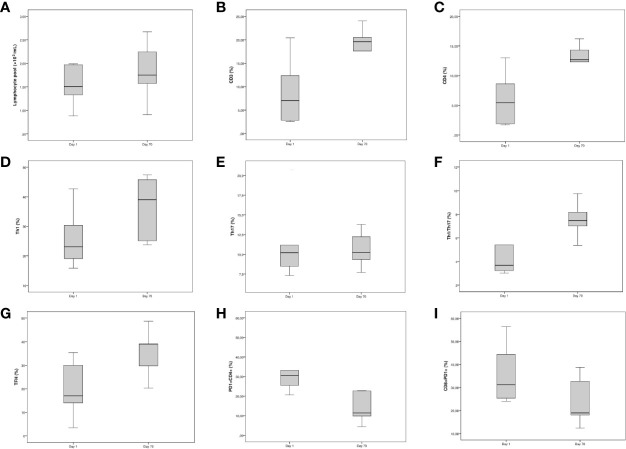
Longitudinal study of the fraction of Th populations in peripheral blood of non-intensive care unit (ICU) hospitalized patients. The line within the box represents the median, the top and the bottom of the box represents the 75^th^ and 25^th^ percentiles, respectively. **(A–C)** Boxplots for [left] lymphocyte pool (×10−3/ml), [middle] CD3 (%) and [right] CD4 (%). **(D–F)** Boxplots for [left] Th1 (%), [middle] Th17 (%), and [right] Th1/Th17 (%). **(G–I)** Boxplots for [left] TFH (%), [middle] CD4+PD-1+ T cells, and [right] CD8+PD-1+ T cells.

To evaluate the long-term impact SARS-CoV2 infection on T cell populations, we tested seven COVID-19 patients 10 weeks after hospital admission using flow cytometry. Notably, when compared to day 1, we observed a significative reduction in PD-1+CD4+T cells and an increase of TFH. Although not statistically significant due to the low number of cases tested, we observed a noticeable recovery of T cells, CD4+ T cells, Th1, and Th1/Th17 cells (**Figure 2**).

We also compared the levels of lymphocyte subpopulations on day 70, with those of healthy donors. Recovered patients showed significantly lower levels of PD-1+CD4+ T cells, TIGIT+CD4+ T cells, and PD-1+CD8+ T cells, while on the other hand, the levels of TFH cells were significantly higher (data not shown).

### COVID-19 Infection Is Associated With Changes in Biochemical and Immunological Parameters

**Figure 3:**
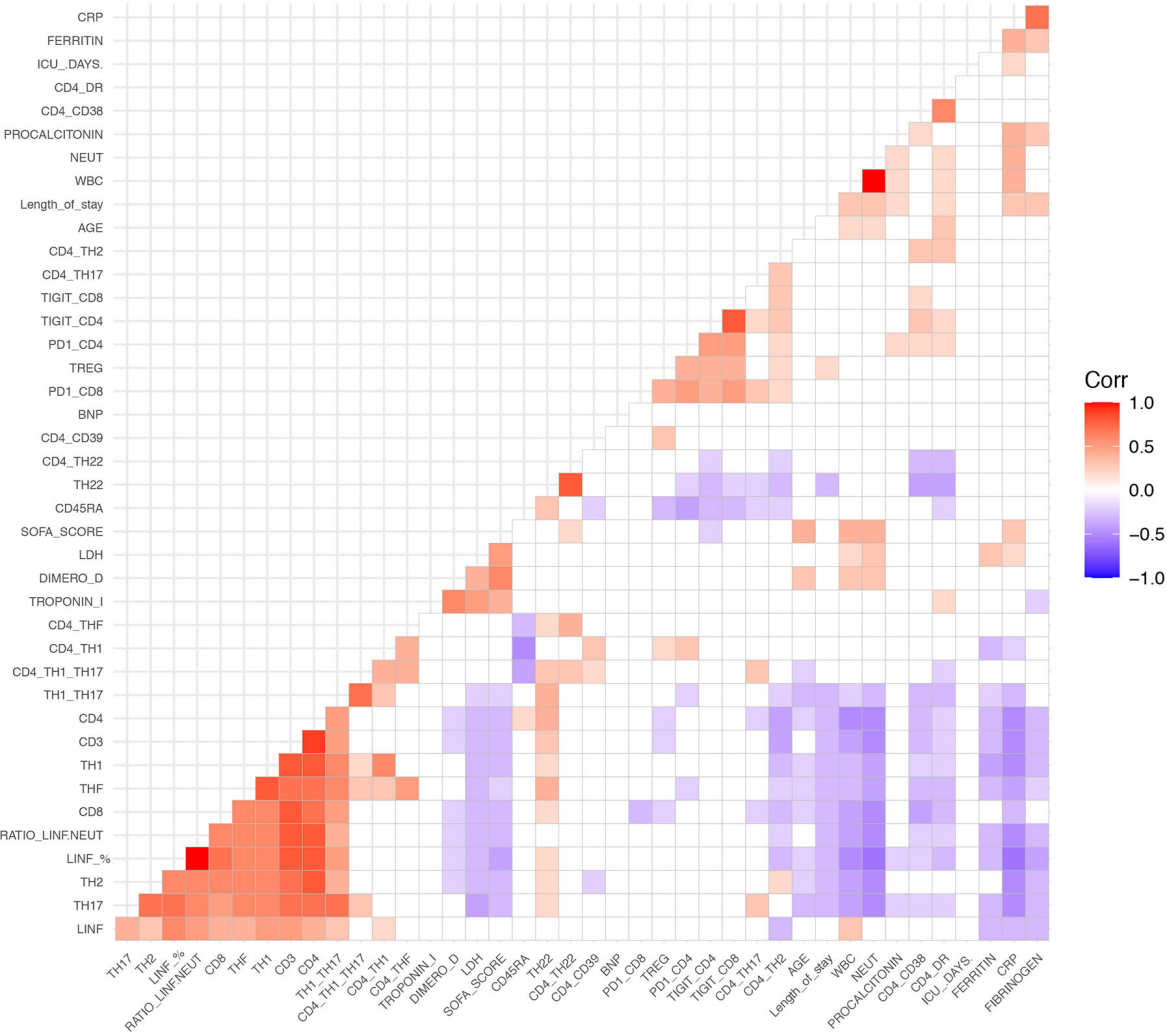
Spearman correlation heatmap of all measured cell populations, biochemical parameters and clinical parameters. Red indicates a positive correlation and blue represent a negative correlation. Non-significant correlations are left blank.

We next performed a correlation mapping to evaluate potential associations between clinical features, biochemical parameters, and leukocyte populations (**Figure 3**). We observed a negative correlation between biochemical inflammatory parameters (ferritin, fibrinogen, CRP, D-dimer, LDH) with the percentage of lymphocytes as well as with CD3, CD4, Th1, Th17, Th1/Th17, and THF. In contrast, the above inflammatory parameters showed a positive correlation with the neutrophils counts.

Interestingly, we found an inverse correlation between lymphocytes and neutrophils. Age exhibited a negative correlation with lymphocyte counts CD4, CD8, Th1, Th17, Th1/Th17, TFH; and a positive correlation with whole blood count and neutrophils.

The expression of the activation markers HLA-DR and CD38 correlated negatively with the percentage of CD3, CD4, CD8, Th17, Th1/Th17, TFH, and Th22 cells, while they correlated positively with CD4+TIGIT+ and CD4+PD-1+ cells. HLA-DR+CD4+ cells was positively correlated with length of hospitalization and with neutrophil counts.

The naïve T cell marker CD45RA had a strong negative correlation with Th1 cells and Tregs, as well as with exhaustion markers (CD4+TIGIT+, CD4+PD-1+, CD8+TIGIT+, CD8+PD-1+, and CD4+CD38+).

Length of stay was negatively correlated with CD3, CD4, CD8, Th17, Th1/Th17, and Th22. On the other hand, length of stay showed a positive correlation with neutrophils, Tregs, HLA-DR+CD4+, fibrinogen, CRP, and procalcitonin.

The SOFA score had a negative correlation with lymphocytes and T cells subpopulations, and a positive correlation with age, length of hospitalization, neutrophil counts, and biochemical inflammatory parameters (LDH, D-dimer, troponin I, and CRP).

In a further step, we evaluated the impact of neutrophilia and lymphopenia on the clinical outcome. We compared several biochemical and immunological parameters across patients with neutrophilia (n=25), and those with neutrophils in the normal range (n=75); besides across patients with lymphopenia (n=49), and those with lymphocytes in the normal range (n=51). Our results demonstrate that neutrophilic patients show an increase in the percentage of Tregs, CD38+CD4+ T cells, and HLA-DR+CD4+ T cells. Furthermore, patients with neutrophilia had increased levels of CRP, LDH, fibrinogen, D-dimer, procalcitonin, and troponin I, and reduced concentration of ferritin and BNP. The SOFA score was significantly elevated in neutrophilic patients (**Table 2**). In regard to the impact of lymphopenia, lymphopenic patients had increased Th2, CD38+CD4+ T cells, HLA-DR+CD4+ T cells, and Tregs (**Table 3**). In addition, patients with lymphopenia had increased mean levels of ferritin, CRP, fibrinogen, D dimer, procalcitonin, and troponin I (**Table 3**).

**Table 2:** Differences in diverse markers between patients with neutrophilia and no neutrophilia among non-intensive care unit (ICU) hospitalized patients.

	No Neutrophilia (n=75)		Neutrophilia (n=25)		P
	Median	(Q_1_–Q_3_)	Median	(Q_1_–Q_3_)	
WBC (×10^−3^/ml)	6,010	(4,540–7,160)	14,000	(11,460–20,090)	1.73x10^−13^_*_
Neutrophil pool (×10^−3^/ml)	4,080	(3,203–5,660)	11,490	(9,440–17,590)	8.46x10^−14^_*_
Lymphocyte pool (×10^−3^/ml)	1,130	(760–1,550)	1,120	(850–1,820)	n.s
T cells (×10^−3^/ml)	858	(546–1,153)	889	(684–1,341)	n.s
T-CD4+	385	(280–562)	398	(206–598)	n.s
T-CD8+	172	(101–319)	181	(102–282)	n.s
**CD4 T lymphocytes (%)**					
Th1	23.77	(17.17–31.63)	26.38	(18.49–30.34)	n.s
Th17	10	(7–13)	10	(9–14)	n.s
Th1/Th17	5.36	(3.77–7.42)	6.38	(4.8–8.55)	n.s
Th22	1.13	(0.77–1.739)	1.41	(0.64–2.12)	n.s
Th2	7.25	(5.71–8.77)	7.69	(6.54–8.92)	n.s
TFH	18.37	(13.09–25)	18.72	(11.7–25.53)	n.s
CD39+ CD4+	4.91	(1.81–7.5)	3.67	(2.47–13.01)	n.s
CD38+ CD4+	4.52	(3.28–6.2)	5.66	(4.06–8.11)	**0.016**
HLA-DR+CD4+	12.09	(9–18.31)	19.74	(12.08–24.03)	0.004*
Tregs	5.67	(4.72–7.64)	7.46	(5.59–9.31)	**0.026**
CD45RA+	33.37	(18.11–43.77)	34.44	(22.62–40.95)	n.s
PD-1+ CD4+	25.44	(17.6–37.05)	31.82	(20.7–40.6)	n.s
TIGIT+CD4+	12.75	(7.37–17.15)	14.55	(12.5–20.8)	n.s
**CD8 T lymphocytes (%)**					
PD-1+ CD8+	32.38	(19.24–41.4)	34.43	(20.26–44.4)	n.s
TIGIT+ CD8+	25.60	(15.03–34.2)	35.30	(14–46.4)	n.s
**Biochemical parameters**					
Ferritin (ng/ml)	509.40	(276.3–908)	479.60	(306.6–689.8)	**4.14x10^−4^**
CRP mg/dl	50.70	(13.6–116.5)	129.50	(88–180.2)	0.001*
LDH (U/L)	322.00	(272–391)	381.50	(303.5–620.5)	n.s
Fibrinogen (mg/dl)	553	(465–709)	723	(589–773)	0.014*
D dimer (mg/L)	0.87	(0.47–1.51)	2.44	(1.39–5.72)	1.66x10^−4^*
Procalcitonin (ng/ml)	0.13	(0.06–0.33)	0.75	(0.42–3.83)	0.014*
Troponin I(pg/ml)	13.40	(5.7–30.8)	93.90	(20.5–376.9)	0.003*
BNP (pg/ml)	181.10	(45.6–276.3)	65.50	(39–170.4)	n.s
SOFA score	1	(0–3)	3	(1.5–6.5)	0.001*

P-value was considered significant only when it was smaller than 0.05. Q_1_. Percentile 25; Q_3_. Percentile 75. *Statistical analysis was evaluated by Mann–Whitney U test.

WBC, white blood cells; n.s, not significant. P values indicated in bold are statistically significant.

**Table 3:** Differences in diverse markers between patients with and without lymphopenia non-intensive care unit (ICU) hospitalized patients.

	No lymphopenia (n=51)		Lymphopenia (n=49)		P
	Median	(Q_1_–Q_3_)	Median	(Q_1_–Q_3_)	
WBC (×10^−3^/ml)	7,160.00	(5,540–11,000)	6,150.00	(4,420–9,160)	**0.023**
Neutrophil pool (×10^−3^/ml)	4,930.00	(3,440–7,910)	4,730.00	(3,340–7,270)	n.s
Lymphocyte pool (×10^−3^/ml)	1,570.00	(1,330–1,970)	760.00	(580–900)	6.92x10^−18^*
T cells (×10^−3^/ml)	1,153	(1,018–1,478)	555	(372–725)	2.14x10^−13^*
T-CD4+	531	(418–692)	284	(177–358)	1.46x10^−10^*
T-CD8+	274.75	(169–380)	112.55	(74–173)	7.96x10^−08^*
**CD4 T lymphocytes (%)**					
Th1	26	(17.94–32.24)	22	(17.17–30.71)	n.s
Th17	10	(8–14)	10	(8–13)	
Th1/Th17	6	(4.09–8.55)	6	(3.8–6.81)	n.s
Th22	1	(0.77–1.76)	1	(0.68–1.78)	n.s
Th2	7	(5.17–8.21)	8	(6.02–9.68)	**0.040**
TFH	20	(13.98–26.81)	17	(11.7–24.89)	n.s
CD39+ CD4+	5.32	(1.98–7.47)	3.74	(1.77–10.23)	n.s
CD38+ CD4+	3.95	(3.24–5.47)	5.66	(4.06–7.48)	0.003*
HLA-DR+ CD4+	11.36	(8.92–18.31)	14.83	(10.12–21.89)	**0.046**
Tregs	5.59	(4.72–6.71)	7.08	(5.22–8.88)	**0.037**
CD45RA+	36.76	(21.73–42.41)	28.59	(18.11–43.59)	n.s
PD-1+ CD4+	25.44	(15.3–36.8)	28.20	(20.6–40.6)	n.s
TIGIT+CD4+	12.80	(9.7–17.15)	13.85	(8.5–18.4)	n.s
**CD8 T lymphocytes (%)**					
PD-1+ CD8+	32.61	(21.8–42.9)	32.37	(17.4–41.5)	n.s
TIGIT+CD8+	30.80	(22.9–44.9)	24.00	(12.8–33.01)	n.s
**Biochemical parameters**					
Ferritin (ng/ml)	509.40	(219.6–767.9)	504.70	(288.4–1,089.8)	**4.14x10^−4^**
CPR mg/dl	40.6	(8–116.2)	97.1	(30.5–161.2)	0.008*
LDH (U/L)	305.00	(266–381)	370.00	(294–429)	n.s
Fibrinogen (mg/dl)	558.00	(452–671)	649.00	(515–790)	0.014*
D dimer (mg/L)	0.87	(0.47–1.755)	1.39	(0.66–2.46)	1.66x10^−4^*
Procalcitonin (ng/ml)	0.38	(0.11–0.745)	.27	(0.085–2.16)	0.014*
Troponin I(pg/ml)	12.45	(5.55–25.8)	21.80	(7.7–117.3)	0.003*
BNP (pg/ml)	96.900	(39–200)	174.200	(53.4–389.35)	n.s
SOFA score	1	(0–3)	1	(0.5–3)	0.001*

P-value was considered significant only when it was smaller than 0.05.

Q_1_. Percentile 25; Q_3_. Percentile 75.

*Statistical analysis was evaluated by Mann–Whitney U test.

n.s, not significant.

P values indicated in bold are statistically significant.

### Differences in Biochemical Parameters and Lymphocyte Subpopulations Between Survivors and Non-Survivors

Among the 100 non-ICU hospitalized patients diagnosed with COVID-19, 21 died in the hospital (21%). As shown in **Table 3**, non-survivors showed at admission higher serum levels of troponin I, CRP, D-dimer, LDH, and BNP compared to survivors.

In regard to lymphocytes, the percentages of lymphocytes, T cells, CD4+ T cells, and CD8+ T cells were significantly higher in surviving patients, and the ratio of neutrophils:lymphocytes was considerably elevated in non-survivors (**Table 4**). Moreover, the percentage of HLA-DR+CD4+, PD-1+CD4+ and PD-1+CD8+ T cells was notably raised in the group of patients that died (**Table 4**).

**Table 4:** Comorbidities and differences in several markers between survivors and deceased among non-intensive care unit (ICU) hospitalized patients.

	Deceased (n=21)	Survivors (n=79)	P
Age	87 (78–90)	70 (56–85))	**1.65x10^−4^**
Male	11 (52.4%)	38 (48.1%)	n.s
Female	10 (47.6%)	41 (51.9%)	n.s
WBC (×10^−3^/ml)	7,840 (6,220–10,760)	6,430 (4,770–9,450)	n.s
Neutrophil pool (×10^−3^/ml)	6,480 (4,530–9,440)	4,620 (3,350–7,290)	n.s
Lymphocyte pool (×10^−3^/ml)	760 (510–1,290)	1,240 (840–1,680)	**0.007**
Neutrophils:lymphocytes	7.22 (3.9–16.4)	4.08 (2–68–6.65)	**0.006**
T cells (×10^−3^/ml)	635 (361–1,086)	889 (625–1,248)	n.s
T-CD4+	312 (204–467)	397 (284–584)	n.s
T-CD8+	173 (74–257)	172 (104–315)	n.s
CD3 (%)	6.83 (2.35–10.33)	9.17 (5.54–13.97)	**0.020**
CD4 (%)	4.30 (1.68–7.18)	5.81 (3.46–9.17)	**0.030**
CD8 (%)	1.50 (0.74–2.92)	2.46 (1.38–4.42)	**0.043**
**CD4 T lymphocytes (%)**			
Th1	24.92 (20–28.96)	24.58 (17–32.7)	n.s
Th17	10.02 (8.84–14.04)	9.85 (7.48–13.41)	n.s
Th1/Th17	5.32 (3.28–7)	5.79 (3.97–7.77)	n.s
Th22	1.37 (0.68–2.02)	1.13 (0.76–1.77)	n.s
Th2	6.54 (4.66–9.68)	7.47 (5.75–8.64)	n.s
TFH	13.7 (10.4–23.42)	20.09 (13.68–25.53)	n.s
CD39+ CD4+	4.74 (2.57–13.88)	4.72 (1.81–7.47)	n.s
CD38+ CD4+	5.64 (4.06–7.97)	4.54 (3.48–6.2)	n.s
HLA-DR+ CD4+	19.74 (13.21–25.02)	12.30 (9.04–18.18)	**0.005**
Tregs	5.7 (5.05–9.03)	6.21 (4.9–7.89)	n.s
CD45RA+	28.6 (15.41–38.25)	34.87 (21.47–44.05)	n.s
PD-1+ CD4+	40.40 (25.42–45.05)	23.50 (16.20–36.30)	**0.004**
TIGIT+CD4+	12.4 (5.9–21.5)	13.85 (9.7–18)	n.s
**CD8 T lymphocytes (%)**			
PD-1+ CD8+	40.30 (25.05–51.97)	30.15 (18.75–41.03)	0.0040*
TIGIT+CD8+	30.3 (15.03–42.7)	28.7 (14.38–38.8)	n.s
**Biochemical parameters**			
Ferritin (ng/ml)	677.2 (311.2–1,186.8)	494.1 (239.6–871.9)	n.s
CRP mg/dl	108.80 (40.85–180.65)	57.60 (13.60–126.10))	0.022*
LDH (U/L)	388 (325–544)	313 (267–392)	**0.006**
Fibrinogen (mg/dl)	649 (540–809)	581.5 (492–709)	n.s
D dimer (mg/L)	1.50 (0.97–5.76)	0.87 (0.47–2.12))	**0.029**
Procalcitonin (ng/ml)	0.39 (0.09–3.83)	0.3 (0.06–0.63)	n.s
Troponin I (pg/ml)	85 (17.05–320.13)	12.9 (5.1–30.9)	**0.001**
BNP (pg/ml)	845.00 (161.40–1,575.20)	81.2 (34.7–200)	n.s
SOFA score	3 (1.5–6.5)	81.20 (34.53–202.60)	**0.007**
**Comorbidities**	****	****	****
Hypertension	43 (54.4%)	16 (76,2%)	n.s**
DM	17 (21.5%)	6 (28,6%)	n.s**
CKD	10 (12.7%)	6 (28,6%)	n.s**
CVD	9 (11.4%)	5 (23,8%)	n.s**
Smoker	12 (15.2%)	2 (9.5%)	n.s**
Overweight/obesity	5 (6.3%)	1 (4.8%)	n.s**
MI	8 (10.1%)	4 (19.0%)	n.s**
HF	3 (3.8%)	7 (33.3%)	0.001**
COPD	7 (8.9%)	2 (9.5%)	n.s**
Asthma	5 (6.3%)	1 (4.8%)	n.s**
PAD	5 (6.3%)	1 (4.8%)	n.s**

P-value was considered significant only when it was smaller than 0.05.

*Statistical analysis was evaluated by Mann–Whitney U test. **Statistical analysis was evaluated by Fisher exact test.

n.s, not significant.

COPD, chronic obstructive pulmonary disease; CKD, chronic kidney disease; CVD, cerebrovascular disease; DM, diabetes mellitus; ET, endotracheal tube; HF; heart failure; ICU, intensive care unit; NIMV, non-invasive mechanical ventilation; MI, myocardial infarction; PAD, peripheral artery disease; peptic ulcer disease.

P values indicated in bold are statistically significant.

With respect to comorbidities, heart failure is significantly more frequent in non survivors (**Table 4**). Although the differences are not statistically significant, hypertension, diabetes mellitus, chronic kidney disease, and cerebrovascular disease are also more frequent in non-survivors.

### Receiver Operating Characteristic Analysis

To examine the diagnostic usefulness of the measured parameters for the prediction of death, we compared sensitivities and specificities at optimal cut-off values determined by ROC analysis.

According to the AUC results obtained, an optimal cut-off value of <13.7% lymphocytes showed a 71% sensibility and a 68% specificity for the prediction of a fatal outcome. HLA-DR+CD4+ (cut-off value >16.2%), and PD-1+CD4+ (cut-off value >24.85%) could also predict death with a sensibility of 71 and 86%, respectively; and a specificity of 68 and 54%, respectively.

**Figure 4:**
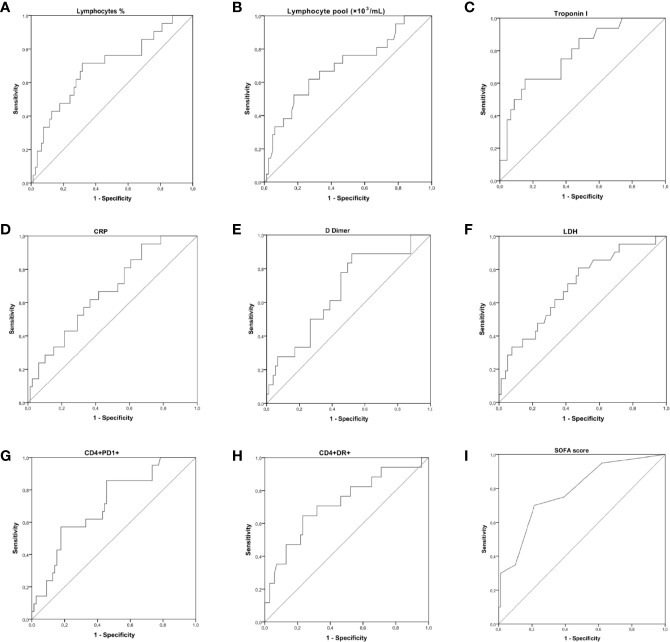
Receiver operating characteristic (ROC) curve showing the accuracy of measuring lymphocytes, PD-1+CD4+ T cells, HLA-DR+CD4+ T cells, troponin I, CRP, D-dimer, lactate dehydrogenase (LDH), and the score Sequential Organ Failure Assessment Score (SOFA) at hospital admission for the prediction of a fatal outcome. The true positive rate (sensitivity) is plotted in function of the false positive rate (1-specificity). The area under the ROC curve (AUC) is a measure of the predictive efficiency of the analyzed parameters to distinguish between the different outcomes. **(A–C)** ROC curves performed for [left] lymphocytes (%), [middle] lymphocyte pool (×10^−3^/ml), and [right] Troponin I. **(D–F)** ROC curves performed for [left] CRP, [middle] D dimer, and [right] LDH. **(G–I)** ROC curves performed for [left] CD4+PD-1+ T cells, [middle] HLA-DR+CD4+ T cells, and [right] the SOFA score.

Death of non-ICU hospitalized patients could be predicted by measuring serum concentrations at hospital admission of troponin I, CRP, D-dimer, and LDH. Our results also indicate that an elevated SOFA score at hospital admission is associated with an unfavorable prognosis. The curves are shown in **Figure 4**. The AUC and optimal thresholds of each risk or protection factor can be found in **Table 5**.

**Table 5:** Receiver operating characteristic (ROC) curves of biochemical and cytometry markers measured at hospital admission for prediction of death.

Predictive marker and cutoff value	AUC	Sensibility (%)	Specificity (%)
Lymphocytes (<13.7%)	0.69	71	68
Lymphocytes (<930×10^−3^/ml)	0.69	67	67
Troponin I (>19.15 pg/ml)	0.78	75	67
CRP (>83.3 mg/L)	0.67	67	57
D-Dimer (>0.85 mg/L)	0.67	89	48
LDH (>342 U/L)	0.70	67	62
PD-1+CD4+ (>24.85%)	0.71	86	54
HLA-DR+CD4+ (>16.2%)	0.72	71	68
SOFA score (>3)	0.78	70	79

AUC, area under the curve.

## Discussion

SARS-CoV-2 infection produces an immune disorder promoted by lymphopenia that mainly affects T lymphocytes. So far, the lymphopenia has been the main finding that may explain the inadequate immune response to SARS-CoV-2. Recently, variations have been described in populations of memory, naïve and effector T lymphocytes, as well as different markers of activation and exhaustion, both on CD4 and CD8 T lymphocytes (21). In our study, we have performed a comprehensive analysis of the Th cell component and CD8 T cells. In addition, we have analyzed the activation status and the appearance of T cell exhaustion markers. Our data reveal a profound impairment of T cell immunity, which can be explained by four different reasons: 1—important lymphopenia. 2—The highest proportion of cells in a non-functional effector state (naïve T lymphocytes), suggesting a persistent hypoactivation of the immune system. 3—An important reduction in the proportion of Th1 cells. 4—An insufficient activation measured by the expression of HLA-DR and CD38, which in many cases was similar to that observed in the control group.

Lymphopenia affects T lymphocytes, showing significant differences between non-ICU hospitalized patients and ICU hospitalized patients, compared to the asymptomatic recovered donors and healthy controls, with the lowest percentage of T lymphocytes in the ICU group. Lymphopenia affected mainly the populations of CD4 T lymphocytes, with a reduction in absolute numbers of the subpopulations Th1, Th2, Th17, Th1/Th17, Th22, TFH cells, and Tregs, highlighting an approximate 10% reduction in the proportion of Th1 cells. This decrease in the percentages of effector T lymphocytes can be explained by the higher frequency of naïve cells, Th0 (CD45RA +, CXCR3-, CCR6-, CCR10-, CCR4-). This increase in naïve T cells is observed in both the hospitalized patients and ICU patients, and has been referred to in other studies (22). We believe that it can be explained by a regenerative process in response to lymphopenia. Alternatively, it could correspond to a block in the complete stimulation carried out by dendritic cells. In this sense, a dysfunctional activation of dendritic cells has recently been observed, which would result in apoptosis and depletion of T lymphocytes (23).

Lymphopenia has been revealed in numerous studies on this disease, but the causes that produce it are not yet fully clarified and may be due to direct infection of lymphocytes or suppression of bone marrow by the antiviral response. The studies have been postulated from cytopathic effects by the virus (24), which have been questioned (25), to metabolic disorders (26). From an immunological point of view, the lymphopenia could depend on the possible dysfunctional activation of dendritic cells already mentioned (23) and the high concentration of cytokines such as TNF-α, IL-6, and IL-10, which act as negative regulators of the proliferation and survival of T lymphocytes (27). In this sense, we detected an inverse correlation between lymphocyte and neutrophil counts in COVID-19 patients. It is therefore very possible that this lymphopenia is caused by factors triggered during the exacerbation of the innate response. Hence the close association found between lymphopenia and the biochemical parameters analyzed (**Table 3**).

The low frequency of the cellular component Th1 is the main finding of this study and can negatively affect in the immune response against SARS-CoV-2 at various levels. Th1 cells are of vital importance in the elimination of intracellular microorganisms such as mycobacteria and viruses. Its reduction, as we see in COVID-19 patients, can have serious consequences for the control of the SARS-CoV-2 infection and its resolution. Th1 lymphocytes, through the production of the cytokines IL-2 and IFN-γ, participate in the activation, proliferation and differentiation of cytotoxic T lymphocytes (CTLs) and induction of cellular cytotoxicity of virus-infected cells (28). Furthermore, unlike SARS patients, patients with COVID-19 also have elevated levels of Th2 cell-secreted cytokines (such as IL-4 and IL-10), which inhibit the inflammatory Th1 responses (13). The Th1 deficiency would lead to a decrease in the number of active CTLs and therefore a poor immune response to the viral infection. Other studies, some published during the evaluation of our work, show similar findings (29–31). However, these studies are based on fewer cases and focus on IFN-γ-producing cells, or naïve/effector-memory cells. Furthermore, these studies, although interesting, do not distinguish between Th1 and Th1/Th17 cells and do not include a comprehensive analysis of additional functional Th-subtypes. Interestingly, a reduced Th1-type specific immune response (i.e., a lower proportion of IFN-γ secreting cells) against different SARS-CoV-2 antigens was observed and was related with age/comorbidity (32). Finally, data by Roncati and colleagues, suggest that the “moonlighting protein” CD26/DPP4 could explain the Th1 immune lockdown observed in severe cases of COVID-19 (33).

We show that there is a low activation (HLA-DR, CD38) of CD8 cells, which is probably also related to the deficiency in the Th1 population. Finally, the decrease in Th1 lymphocytes can affect the development of antibodies (34). In this sense, it is known that human coronavirus infections occasionally fails to generate protective immunity due to a poor adaptive immune response (35–38). This may be due to an insufficient durability or magnitude of the T cell response as the production of neutralizing antibodies is dependent on the T cell response (39, 40). In agreement, Grifoni and colleagues recently showed that the antibody response to SARS-CoV-2 correlated positively with the magnitude of the Th1 response (41).

To further investigate the relationship between immune responses and COVID-19 disease severity, we used death as a marker of severity. In regard to lymphocytes, the percentage of lymphocytes, T cells, CD4+ T cells, and CD8+ T cells was significantly elevated in survivors (**Table 5**). Moreover, the percentages of HLA-DR+CD4+, PD-1+CD4+, and PD-1+CD8+ T cells were notably raised in the group of patients that died (**Table 5**). PD-1 is a marker of exhausted T cells and is induced in response to continuous stimulation as occurs in chronic infections and cancer (42). In the case of SARS-CoV-2, the virus could be persistently stimulating T lymphocytes, inducing the exhausted state (27).

With regard to the expression of HLA-DR on CD4 and CD8 lymphocytes, it should be noted that in the large majority of patients there was either no or only a minimally significant increase in this marker which is in agreement with Mathew et al. (21). These authors show that the immune response is quite diverse and thus, while a subgroup of patients had T cell activation characteristic of acute viral infection another subgroup had lymphocyte activation comparable to uninfected subjects. This lack of activation contrasts with that observed in the course of other viral infections, which is very prolonged and stable over time (43–45). However, it should be noted that patients with a significant increase in HLA-DR exhibited a more aggressive disease course (**Figure 1B**). Interestingly, we found that COVID-19 patients with neutrophilia, lymphopenia and a more pronounced alteration in the biochemical parameters associated to inflammation had a higher percentage of CD4+ T cells with expression of activation markers HLA-DR and CD38. It is possible that the appearance of HLA-DR in patients with more intense lymphopenia could be due to an expression induced by inflammatory cytokines and is therefore not indicative of antigen-specific activation. It is therefore possible that the expression of HLA-DR is a consequence of dysregulated inflammation and the recruitment of inflammatory myeloid cells (21) and, hence its observation in the context of neutrophilia (**Table 2**). The increased inflammatory state induces the generation of neutrophils through the production of G-CSF, the regulation of the expression of chemoattractants and the activity of neutrophils (46). The positive relationship found between neutrophils and acute phase proteins can be explained too by the inflammatory state (47). The production of acute phase proteins such as ferritin and CRP, in addition to affecting the balance between the pro and anticoagulative pathways (increasing the D-dimer) (48, 49), can induce apoptosis in lymphocytes (13, 50). The study of ROC curves showed that troponin I, CRP, D-dimer, and LDH concentrations at hospital admission can be used as predictors of death. Measuring lymphocytes, HLA-DR+CD4+ T cells, or PD-1+CD4+ T cells could also be useful for the prediction of a fatal outcome. Our results also indicate that an elevated SOFA score at hospital admission is associated with an unfavorable prognosis.

Our study has some limitations. Firstly, we have not included a group of PCR-positive patients (asymptomatic or with mild symptoms) that did not require hospitalization. This group could have clarified the apparent contradictory relevance of HLA-DR/CD38 expression on CD4+ T cells. Secondly, considering the obtained results, it would have been informative to measure the levels of IFN-γ in the serum of the patients in order to compare it to the levels of Th1 cells. Furthermore, it would have been interesting to compare the changes in Th1 cells with the levels of viremia.

In conclusion, the decrease of Th1 cells in COVID-19 infection, especially in older patients, is related to the clinical course of disease. Assuming that virus-induced IFN-γ production is essential for the anti-viral response, a profound decrease in the proportion of Th1 cells in combination with the highest levels of cells in a non-functional effector state, represent an unfavorable scenario for COVID-19 patients, especially for those with a strong lymphopenia. Another important finding of this study are the description of parameters associated with a fatal outcome to COVID-19, including a high neutrophil/lymphocyte ratio, expression of PD-1 on CD4+ and CD8+ lymphocytes, and the expression of HLA-DR on CD4+ T cells in patients with a marked lymphopenia. These changes occur in the context of biochemical indicators associated with a clinical inflammatory syndrome.

## Data Availability Statement

The raw data supporting the conclusions of this article will be made available by the authors, without undue reservation.

## Ethics Statement

The studies involving human participants were reviewed and approved by Portal de Ética de la Investigación Biomédica. Junta de Andalucía (Cod. 0766-N-20). The patients/participants provided their written informed consent to participate in this study.

## Author Contributions

FR-C and PJ contributed to the design of the study. JG-B, PJ, FR-C, and AR-N performed the flow cytometry. JG-B and AR-N performed the analysis of the data obtained by cytometry. JG-B and AR-C built the clinical database. AR-N performed the statistical analysis of the data. ML-R and JG-B collected samples from non-ICU hospitalized patients, UCI hospitalized patients, asymptomatic recovered donors, and healthy donors. FR-C, AR-N, JG-B, PJ, and PA wrote the manuscript. AR-C and ML-R contributed to the clinical follow-up of patients. All authors contributed to the article and approved the submitted version.

## Funding

This work was supported by grants from the Instituto de Salud Carlos III co-financed by FEDER funds (European Union) (PI 16/00752) and Junta de Andalucía in Spain (Group CTS-143). PA is supported by the Consejería de Salud, Junta de Andalucía through the contract “Nicolás Monardes” [C-0013-2018]. This study was partially financed by Becton Dickinson.

## Conflict of Interest

The authors declare that the research was conducted in the absence of any commercial or financial relationships that could be construed as a potential conflict of interest.
