# Supplementary material for: Negative Clinical Evolution in COVID-19 Patients Is Frequently Accompanied With an Increased Proportion of Undifferentiated Th Cells and a Strong Underrepresentation of the Th1 Subset
Source: Front Immunol. 2020 Nov 26;11:596553. doi: 10.3389/fimmu.2020.596553 (PMC7726249; doi:10.3389/fimmu.2020.596553)
Supplement: Supplementary file 1 [file Table_1.docx]

Supplementary Material

**Supplementary Table 1 -** Demographical and clinical features of COVID-19 patients.

|  | **Non-ICU hospitalized patients** | **ICU hospitalized patients** | **Asymptomatic recovered**  **donors** |
| --- | --- | --- | --- |
| n | 100 | 17 | 27 |
| Ages (mean range) | 74 (61,0-87,0) | 69 (58,5 71,0) | 43 (34,0 58,0) |
| Males (%) | 49 (49.00%) | 13 (76.5%) | 5 (18.5%) |
| Females (%) | 51 (51.00%) | 4 (23.5%) | 22 (81.5%) |
| NIMV (%) | 2 (2,0%), | 17(100%) |  |
| ET (%) | 2 (2,0%) | 17(100%) |  |
| Deaths (%) | 21 (21%) | 2 (11.8%) |  |
| Length of admission (days) | 8 (6 – 14) | 61 (49.5 – 77.5) |  |
| ICU admissions (%) | 4 (3,9%) |  |  |
| Length of ICU admission (days) | 12,5 (9,8 -15,3) | 41 (32.5 – 50.5) |  |
| **Comorbidities** |  |  |  |
| - Hypertension  - DM  - CKD  - CVD  - Smoker  - MI  - HF  - COPD  - Alzheimer  - Overweight/Obesity  - Asthma  - PAD  - PUD  - Parkinson  - Neoplasms  - Alcoholism | 59 (59.0%)  23 (23.0%)  16 (16.0%)  14 (14.0%)  14 (14.0%)  12 (12.0%)  10 (10.0%)  9 (9.0%)  8 (8%)  6 (6.0%)  6 (6.0%)  6 (6.0%)  4 (4.0%)  2 (2%)  3 (3.0%)  3 (3.0%) | 9 (52.9%)  5 (6 (35.3)  1 (5.9%)  0 (0%)  29.4%)  1 (5.9%)  1 (5.9%)  1 (5.9%)  0 (0%)  2 (11.9)  2 (11.9%)  0 (0%)  1 (5.9%)  0 (0%)  1 (5.9%)  0 (0%) |  |

CD, Cognitive Disorders; COPD, Chronic obstructive pulmonary disease; CKD, Chronic Kidney Disease; CVD, Cerebrovascular Disease; DM, Diabetes Mellitus; ET, endotracheal tube; HF; Heart Failure; ICU, Intensive Care Unit; NIMV, Noninvasive mechanical ventilation; MI, Myocardial infarction; PAD, Peripheral Artery Disease; Peptic Ulcer Disease.

**Supplementary Table 2 -** Mab reagents used for the immunophenotypic characterization.

| **Mab-conjugates** | **Clone** |
| --- | --- |
| CD3-BV421 | SK7 |
| CD4-APC-H7 | CD4-APC-H7 |
| CD8-FITC | SK1 |
| CCR10-PerCP-Cy5.5 | 1B5 |
| CXCR3 (CD183)-PE | 1C6/CXCR3 |
| CXCR5 (CD185)-BB515 | RF8B2 |
| CD194 (CCR4)-PE-Cy7 | 1G1 |
| CD196 (CCR6)-APC | 11A9 |
| TIGIT-BV510 | 741182 |
| PD1 (CD279) PerCP-Cy5.5 | EH12.1 |
| CD39-PE | TU66 |
| HLADR-V500 | G46-6 |
| CD25-PE-Cy7 | 2A3 |
| CD127-AF647 | HIL-7R-M21 |
| CD38-APC-H7 | HB7 |

Abbreviations: AF: alexa fluor; APC: allophycocyanin; BB: brilliant blue 515; BV: brilliant violet, FITC: ﬂuorescein isothiocyanate; PE: phycoerythrin; PerCP-Cy5.5: peridinin chlorophyll protein-cyanine 5.5.

**Supplementary Table 3 -** Immunophenotypic profiles used for the selection of Th subsets.

| Populations of blood CD3+ CD4+ CD45^hi^ T-cells | CD183 | CD185 | CD194 | CD196 | CCR10 |
| --- | --- | --- | --- | --- | --- |
| CD4+ naïve T cells (Th0) | - | - | - | - | - |
| CD4+ Th1 cells | + | - | - | - | - |
| CD4+ Th2 cells | - | - | + | - | - |
| CD4+ Th17 cells | - | - | + | + | - |
| CD4+ Th1/Th17 cells | + | - | - | + | - |
| CD4+ Th22 cells | - | - | + | + | + |
| CD4+ TFH cells | -/+ | + | -/+ | -/+ | - |

Markers used for the identification of the different T cells subsets. CD183: CXCR3; CD185: CXCR5; CD194: CCR4; CD196: CCR6. “-/+”: partial expression.
